# Impact of Dust Storms on Airborne Bacteria, Heavy Metals, and Inflammatory Markers in Asthmatic Patients

**DOI:** 10.1002/mbo3.70109

**Published:** 2025-11-04

**Authors:** Alaa Al‐Husseini, Majid Komijani, Rulla Sabah

**Affiliations:** ^1^ Department of Biology, Faculty of Science Arak University Arak Iran; ^2^ Department of Chemistry, College of Science Mustansiriyah University Baghdad Iraq

**Keywords:** air born bacteria, asthma, droughts, dust, heavy metals, Iraq

## Abstract

Asthma, a chronic bronchial disorder prevalent in children/adolescents, is exacerbated under environmental conditions like dust storms. The current study investigated heavy metal levels, airborne bacteria, and serum IL‐4/IL‐8 in asthmatics during before/after dust storms in Iraq's Al‐Anbar, Baghdad, and Kirkuk provinces. Airborne heavy metals were quantified by ICP‐MS, serum cytokines by ELISA, and bacterial communities via metagenomics. Statistical analysis was performed using GraphPad Prism (*p* < 0.05 significant). ICP‐MS revealed considerably elevated post‐storm concentrations of As, Ag, B, Ba, Co, Hg, Mg, Mn, Ni, Sn, S, Ti, and V. Asthmatic subjects presented with considerably elevated IL‐4 and IL‐8 post‐storm (*p* < 0.05) compared to controls (*p* > 0.05). Metagenomics revealed storm‐induced bacterial alterations: Al‐Anbar contained elevated *Burkholderiaceae*, *Methylophilaceae*, and *Rhodobacteraceae*; Kirkuk contained elevated *Ilumatobacteraceae*, Microbacteriaceae, Burkholderiaceae, and *Rhodobacteraceae*. Baghdad's most prevalent species included *Rhodocyclaceae* (50%), *Burkholderiaceae* (17%), and Arcobacteraceae (4.5%). Al‐Anbar was significantly richer in microbes (Chao1) and more diverse (Shannon) than other regions following the dust storm (*p* < 0.0001). These findings indicate that dust storms raise heavy metals, alter airborne bacteria, and increase inflammatory cytokines in asthma sufferers, and these emphasize their role in exacerbating asthma in Iraq.

## Graphical abstract

Dust storms in Iraq elevate airborne heavy metals (As, Ag, Ba, and so on) and alter microbial communities, exacerbating allergic inflammation (IL‐4/IL‐8) in asthmatics. Metagenomics reveals storm‐driven shifts in dominant bacteria (e.g., *Burkholderiaceae*, *Rhodocyclaceae*), linking environmental pollution to asthma risk

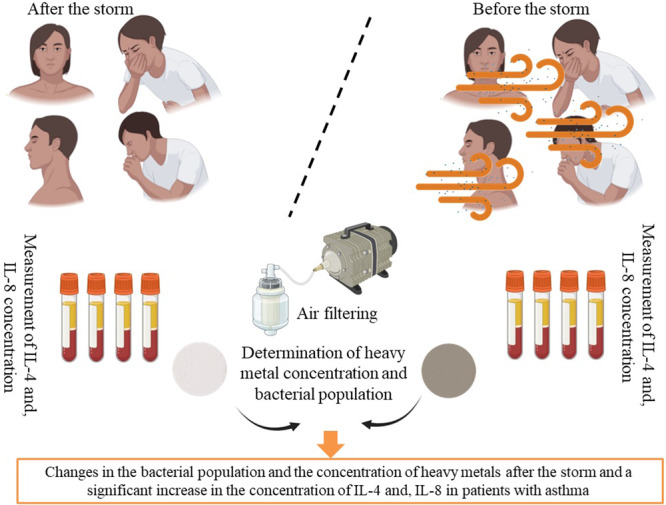

**Abbreviations**
AHR: airway hyperresponsiveness
APCs: antigen‐presenting cells
Cd: cadmium
Cr: chromium
Cu: copper
DCs: dendritic cells
ELISA: enzyme linked immunosorbent assay
Hg: mercury
HRP: horse radish peroxidase
ICP‐MS: inductively Coupled Plasma – Mass Spectrometry
IL‐4: interleukin‐4
IL‐8: interleukin ‐8
ISAAC: The International Study of Asthma and Allergies in Childhood
MHC: major histocompatibility complex
Pb: lead
PCR: polymerase chain reaction
ROS: reactive oxygen species
Th2: T helper type 2
TMB: tetramethylbenzidine
(TNF)‐α: tumor necrosis factor

## 1 Introduction

Asthma is the most common chronic inflammatory lung allergic condition, with heterogeneous endotypes (pathways) causing variable presentation. Two major endotypes are T‐helper type 2 (TH2)‐high asthma, characterized by eosinophilic inflammation and elevated cytokines (IL‐4, IL‐5, IL‐13, IgE), and TH2‐low asthma with neutrophilic or pauci‐granulocytic profiles and corticosteroid‐resistance (Stokes and Casale 2016). With the increasing prevalence of asthma worldwide, enormous healthcare and economic impacts have been imposed on the impacted countries. Most cases of asthma follow a variable course with wheezing triggered by viral infections or by allergen sensitization with varied mechanisms in different individuals (Holgate et al. 2015).

Air pollution from ambient sources, comprised of harmful chemicals, significantly enhances the risk of respiratory death. The largest burden is shouldered by developing nations, with 91% of deaths caused by pollution occurring in low‐ and middle‐income nations, increasingly in Asia and Africa. Air pollution consists of gaseous emissions (NO₂, SO₂, O₃) and particulate matter (PM), vehicle emissions posing a particularly deadly threat to lung functioning. Natural sources contribute, yet industrialization has been the foremost causative factor of diminishing air quality worldwide. Asthma, a condition of chronic inflammatory airway illness occurring in 1%–18% of the global population, has unequivocal relationships with air pollution, and road traffic pollution has been estimated to cause 13% of pediatric cases and exacerbate symptoms in all ages (Tiotiu et al. 2020). Dust storms also worsen these health risks, and research indicates that elevated PM levels during dust storms are linked to increased mortality, particularly due to cardiovascular disease. Barcelona and some areas in Asia have had research conducted with evidence that dust storms increase inflammatory markers, decrease lung function, and worsen asthma. However, findings in Italy, Greece, Kuwait, and Taipei show no statistically significant association between dust exposure and increased mortality or hospitalization due to respiratory causes. Such discrepancies are susceptible to scientific explanation, perhaps as a result of geographic differences across areas in the composition of dust or population susceptibility. Hypothetical mechanisms suggest that fine PM in dust storms is an immune stimulant, with T‐lymphocyte activation in the respiratory tract and leading to chronic inflammation and progressive pulmonary disease. In addition, exposure to PM can induce airway oxidative stress, augment pulmonary inflammation, and augment allergic responses—particularly asthma—through Immunoglobulin E production (Aghababaeian et al. 2021).

The prevalence of asthma in population aged less than 20 years in Middle East countries (Alavinezhad and Boskabady 2018). The results of four studies about the prevalence of asthma in Iraq showed 8.9% prevalence in older and 15.55% in younger children. Environmental pollution, family history of asthma, and exposure to cigarette smoking were recognized as serious risk factors for asthma prevalence in this country (Alsamarai et al. 2009).

In Iraq, The International Study of Asthma and Allergies in Childhood (ISAAC) recorded the prevalence of clinically diagnosed childhood asthma as 16.3% in primary school children Several epidemiological studies on the prevalence of childhood asthma were conducted in different regions of Iraq; the largest one was carried out by Al‐Thamiri et al in Baghdad and involved 3360 primary school children. This is the only study from Iraq that is included in ISAAC (Abood and Al‐Zaubai 2020).

Th2 cells play a critical role in the pathophysiology of Asthma. IL‐4, IL‐5, and IL‐13 cytokines are secreted during the immune responses of these cells. Cytokine IL‐4, with its role in changing the isotype of B cell IgE and positive regulation of FcεRI on mast cells, leads to the release of histamine and other mediators and provides the basis for allergic symptoms and smooth muscle spasms. IL‐5 can play a role in bronchial inflammation by affecting the activation, migration, and accumulation of eosinophils in the airways Athari (2019).

IL‐8 is mainly stimulated by macrophages and other cells and is the main chemoattractant for neutrophils in vitro and in vivo. It forms part of the inflammatory secretions caused by microbial infection in vivo. Other cells that produce IL‐8 include monocytes, neutrophils, basophils, eosinophils, fibroblasts, alveolar macrophages, bronchial epithelial cells, pulmonary microvascular endothelial cells, and airway smooth muscle cells (McElvaney et al. 2020).

The recent droughts in Iraq and the high prevalence of respiratory problems, especially asthma, led us to measure the concentration of heavy metals caused by dust (before and after the dust storm) and drought in the serum of people with asthma. Due to the ability of heavy metals to bind to dust surfaces and their harmful effects on human health and the ecosystem (Zajac 2021), in this study, Five heavy metals, mercury (Hg), Cadmium (Cd), Chromium (Cr), lead (Pb), and copper (Cu) were investigated. Their concentration is measured by the Inductively Coupled Plasma (ICP) spectroscopy method.

In addition, we aimed to survey air dust samples and identify the bacterial strains in the air dust before and after the dust storm using a metagenomic method based on 16sr RNA sequencing (Núñez et al. 2016).

## 2 Materials and Methods

### 2.1 Dust Sampling

#### 2.1.1 Meteorological Information During the Dust Sampling

Several important meteorological factors, such as humidity, temperature, and concentration of M 10 µm in diameter (PM10), were obtained from the meteorological department of the three mentioned provinces. The sampling location was considered the starting point of the backward trajectory examination, and the starting time was 10 UTC (Coordinated Universal Time (UTC). Time in Iraq is given by Arabia Standard Time (AST) (UTC + 03:00). Forty‐eight hours backward trajectories were determined at the height of 500 m to track the source of dust particles during the dust events.

#### 2.1.2 Air Sample Collection

Dust samples from three provinces were collected on the rooftop (30 m height) of the building in Baghdad, Iraq (44°23′1381.49NE, 33° 21′1313.42NE); Al‐Anbar, Iraq, (43°14′ 853.94NE, 33°25′1503.66NE), and Karkuk, Iraq, (44°23′13987NE, 35°27′1668.27NE) before and after the storm. Before the dust storm, the weather was clear with little wind. Dust samples were collected from January to February 2023. The SKC AirChek XR5000 sampling pump (SKC Inc., USA) was used for air filtration. Filters were stored in separate sterile containers for analysis of heavy metals.

Air dust samples were collected on separate filters for ICP‐MS and metagenomic analysis. Filters for heavy metal quantitation were acid‐digested, while filters for analysis of microbial communities were left untreated and processed using DNA preservation protocols.

### 2.2 Measurement of Heavy Metals in Air Dust Samples by ICP‐MS

About 0.1 g of dust sample was added to Teflon containers with a sufficient volume of solids digesting solution containing 4 mL of nitric acid (HNO3), 1.0 mL of Hydrochloric acid (HCL), and 1 mL of hydrofluoric acid (HF), and the samples were digested in the microwave system (Ahmed et al. 2017). After sample digestion, Teflon containers were placed on a ceramic heating plate to dry the samples. The remaining materials were dissolved in 0.14 M nitric acid and then transferred to 50 mL Polyethylene flasks. Finally, The ICP device (Inductively Coupled Plasma Mass Perkin‐Elmer “ELAN9000 USA” Spectrometry) was used for chemical analysis and measurement of heavy metal concentration in dust samples.

### 2.3 Air Dust Metagenomics

In this study, we used the next‐generation sequencing technique based on the 16sr RNA sequencing to identify bacteria in the dust. The 16S rRNA gene sequencing is started with genomic DNA extraction of bacteria from the dust of the indicated areas. The Genomic DNA was extracted using a DNA Extraction kit (DNeasy PowerSoil Kit (QIAGEN Cat No./ID: 12888). Subsequently, the extracted DNA sample was quantified to determine its quantity and quality. Following this, the libraries were prepared based on PCR amplification of the V3‐V4 region as highly variable regions of the 16S rRNA gene (Bartram et al. 2011; Buermans and Den Dunnen 2014) sequencing for bacterial identification. High‐throughput sequencing was conducted on the Illumina NovaSeq. 6000 platform (Illumina, San Diego, CA, USA). Paired‐end sequencing (2 × 250 bp) according to the manufacturer's protocol was conducted. Raw sequence data were quality filtered, trimmed, and chimera removed. Subsequent bioinformatics analysis, such as taxonomic classification and diversity analysis, was performed using CLC Genomics Workbench 22 (Qiagen, Germany) to obtain high‐quality results.

### 2.4 Ethics Committee Approval

According to the necessity of complying with the instructions of the Ministry of Health and Medical Education regarding ethical issues in the field of medical research, the ethical code was received from the specialized committee of Arak University (IR. ARAKU. REC1401.111).

### 2.5 Blood Sample Preparation and ELISA Assay

Blood samples were taken from 100 patients with asthma and 60 healthy people from January to February 2023. The sera were separated and stored in a −20°C freezer until ELISSA assay. Two human ELISA kits (YLA1519HU, and YLA1210HU) were used to measure the IL‐4, IL‐8 levels in the serum sample of Asthmatic and healthy individuals, respectively.

### 2.6 Statistical Analysis

The Paired *t*‐test was used to compare the quantitative values of normal distribution between before and after assessments. The One‐way ANOVA was used to analyze all data obtained. The analysis was performed using the GraphPad version 9.0, and means were compared using the Tukey‐Kramer test to compare Asthma patients and control individuals. A *p*‐value less than 0.05 (typically ≤ 0.05) was considered statistically significant.

## 3 Results

### 3.1 Air Metagenomics Analysis

The composition of airborne bacteria at the phylum, family, and genus levels across three provinces: Al‐Anbar, Baghdad, and Kirkuk was analyzed using the CLC Microbial Genomics Module 23.0.3. To streamline the visualization of OTU (Operational Taxonomic Units) clustering results, samples with low abundance were excluded from the analysis.

#### 3.1.1 Air Metagenomics Analysis in Al‐Anbar

**Figure 1:**
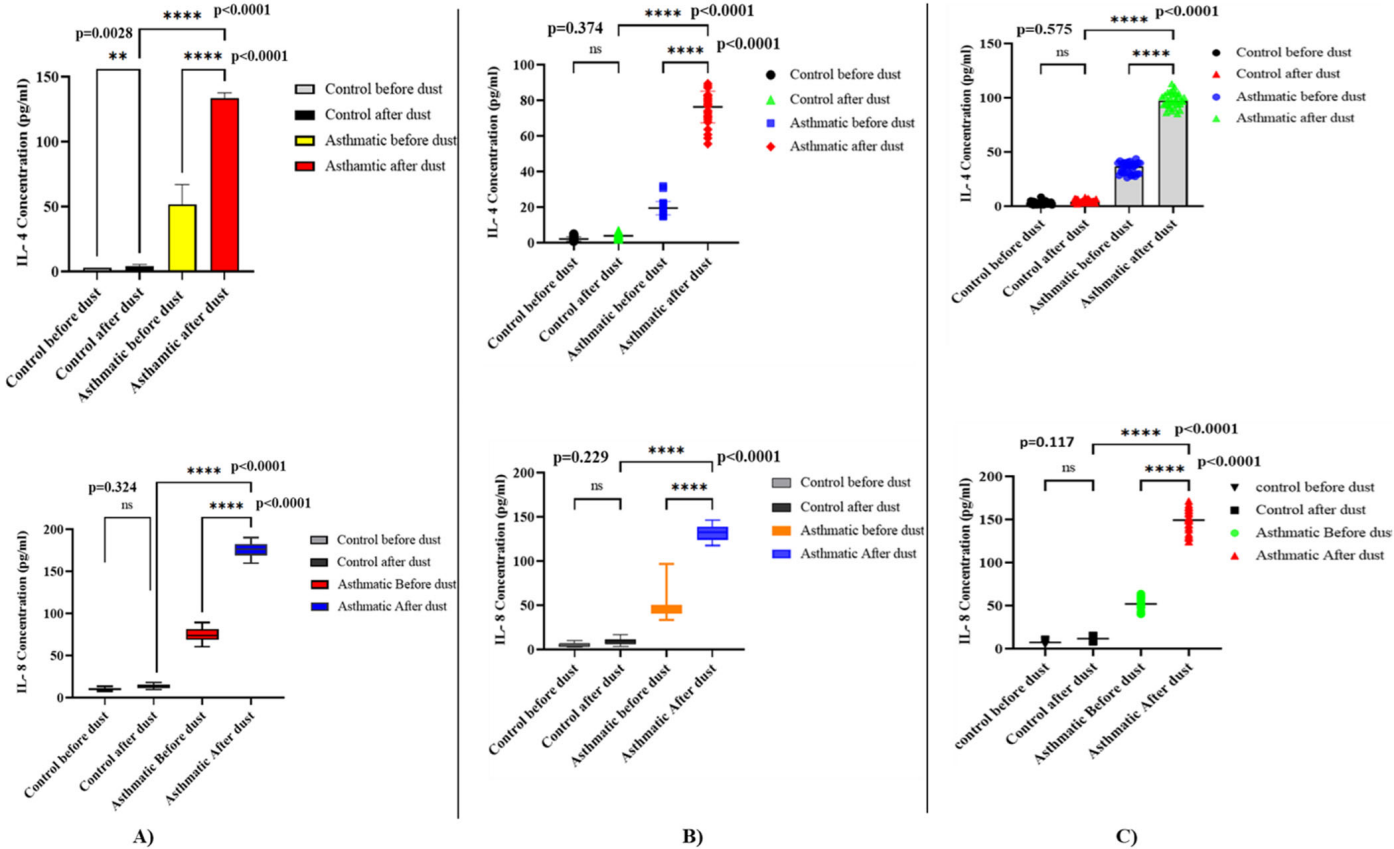
Comparing the IL‐4, and IL‐8 levels in the control and Asthma groups in Anbar (A), Kirkuk (B), and Baghdad (C).

The metagenomic analysis of airborne bacteria in the Al‐Anbar province before a storm, at the phylum level, revealed that Proteobacteria were the most abundant, with 1412 reads, accounting for 47% of the total as shown in Figure 1. Other prominent bacterial phyla included Actinobacteria, with 933 reads (31%), and Bacteroidota, with 246 reads (8%). Following the storm, the abundance of these phyla changed: Proteobacteria increased to 2495 reads (48%), Actinobacteria to 1531 reads (30%), and Bacteroidota to 412 reads (8%).

**Figure 2:**
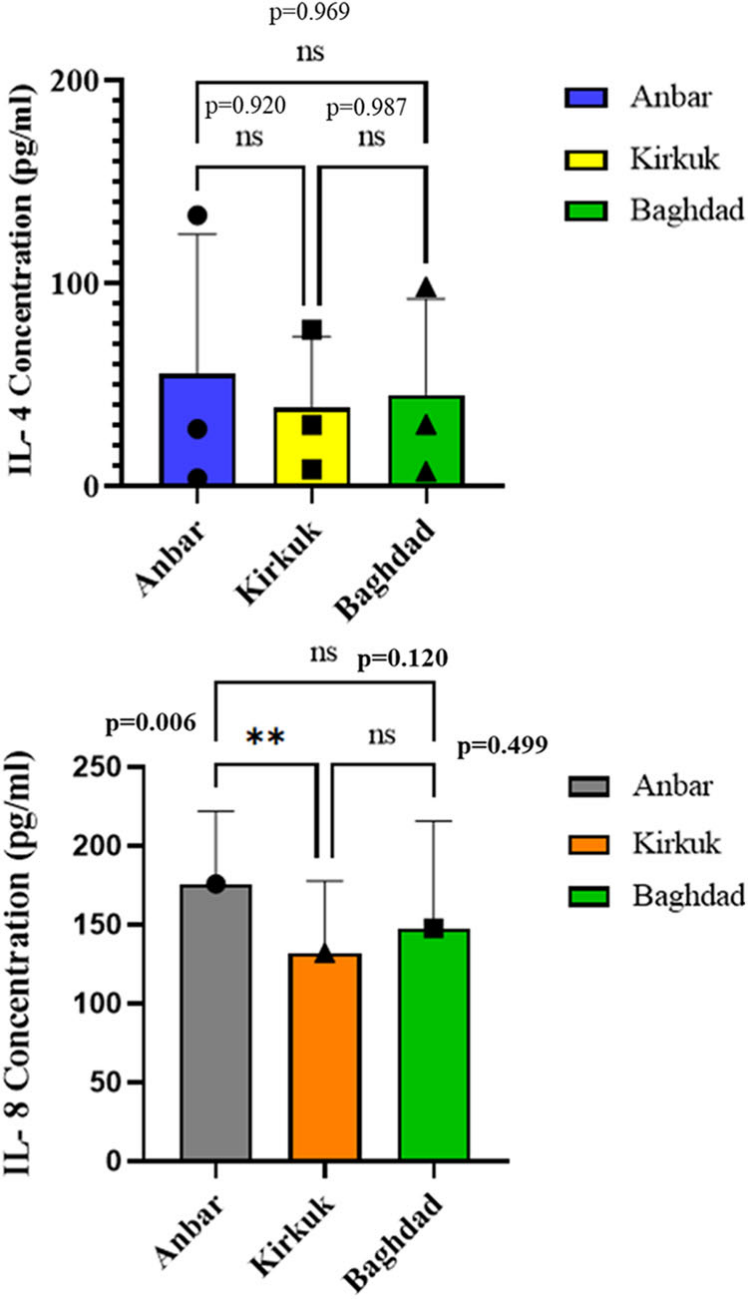
Comparing the IL‐4, and IL‐8 assays after dust storm in Anbar, Kirkuk, and Baghdad.

The paired *t*‐test analysis comparing the bacterial abundance at the phylum level in Al‐Anbar province before and after the storm indicated significant increases in the abundance of Proteobacteria and Actinobacteriota post‐storm. As illustrated in Figure 2A, the *p*‐values for these increases were *p* < 0.0001 for Proteobacteria and *p* = 0.0014 for Actinobacteriota, demonstrating statistically significant differences. In contrast, the analysis showed no significant change in the abundance of the Bacteroidota phylum, with a *p*‐value of *p* = 0.0885.

At the family level, the abundances of *Burkholderiaceae*, *Methylophilaceae*, and *Rhodobacteraceae* significantly increased after the storm compared to before. The paired *t*‐test analysis revealed *p*‐values of *p* = 0.0263 for *Burkholderiaceae*, *p* = 0.0417 for *Methylophilaceae*, and *p* = 0.0374 for *Rhodobacteraceae*, indicating statistically significant differences as shown in Figure 2B.

#### 3.1.2 Air Metagenomics Analysis in Kirkuk

Due to extremely low and non‐countable bacterial reads, the bacterial abundance in the air before the storm in Kirkuk could not be reported. Consequently, only the OTU clustering of the air microbiome after the storm is presented. For statistical analysis, the bacterial abundance before the storm was considered zero. Such very low microbial biomass in pre‐storm samples would most likely be due to the following reasons:

Environmental Conditions Before the Storm: Before the dust storm, the Kirkuk air could have contained much lower PM under stable weather conditions, resulting in little suspension of soil‐ or dust‐particle‐associated bacteria. Microbial numbers in the air in arid and semi‐arid areas such as Kirkuk tend to be highly sensitive to wind‐blown dust dispersion, which was presumably absent or minimal before the storm.

**Figure 3:**
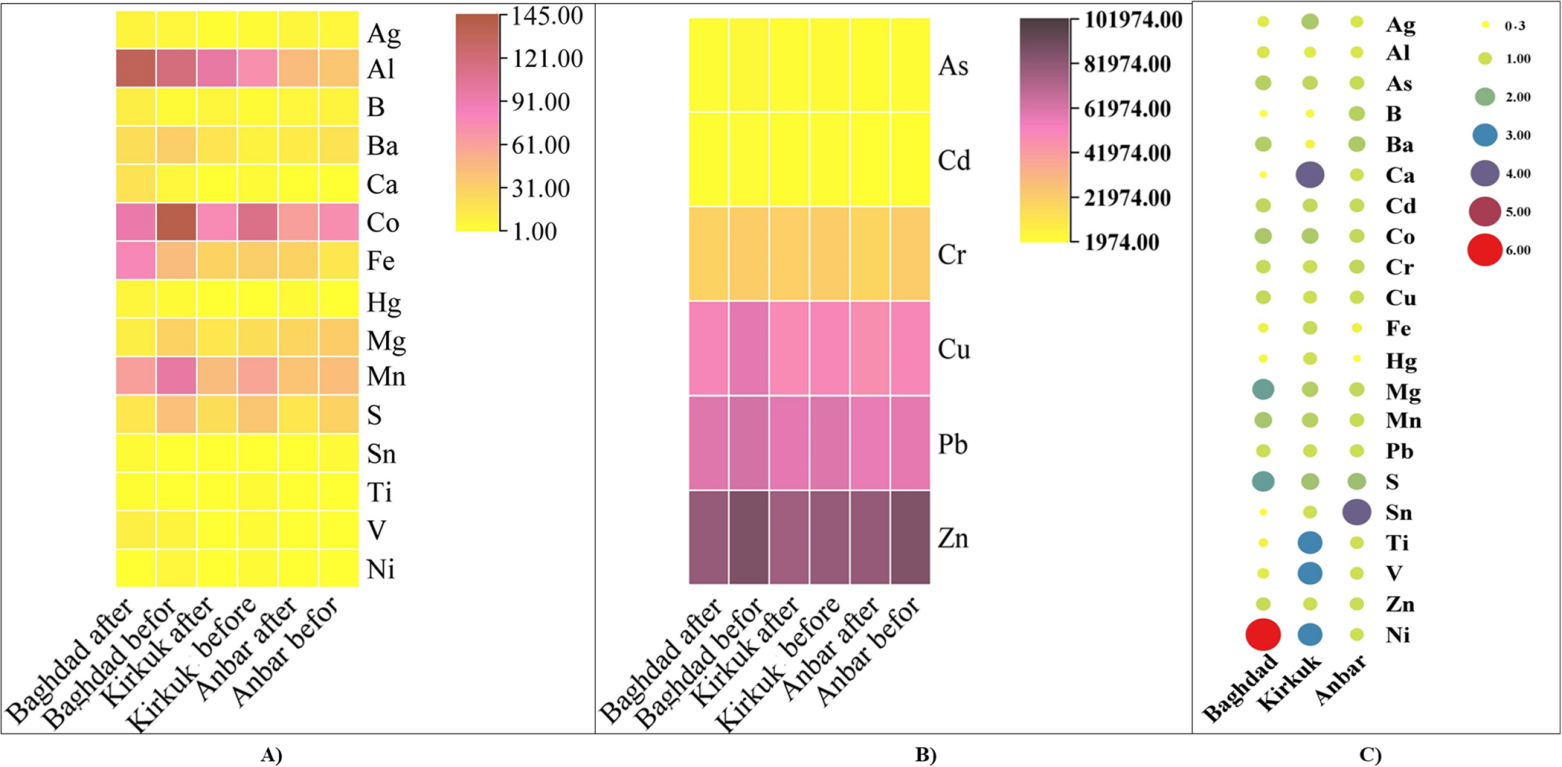
Heat map comparison of the Heavy metal with low (A) and high (B) concentration and the increased ratio of Heavy metals after/before dust in three provinces (C): Low concentration from 1 up to 145 µg/kg dry weight; high concentration from 1974 up to 87,321 µg/kg dry weight.

Technical Limitations in Low‐Biomass Samples: Low‐biomass air sample metagenomic sequencing is technically challenging because the dilute microbial DNA could fall below the thresholds of detection by standard protocols. Filter‐based air sampling tends to lead to DNA loss during extraction, especially in the case of very low microbial loads. The dramatic peak in bacterial reads after the storm (Figure 3A) is in line with the prediction that the dust storm introduced large quantities of soil‐ and environment‐residing microbes into detection.

The paired *t*‐test analysis demonstrated that the abundances of Actinobacteriota, Acidobacteriota, and Proteobacteria significantly increased after the storm, with p‐values of *p* = 0.0306, *p* = 0.0305, and *p* = 0.0490, respectively, as shown in Figure 3A.

As depicted in Figure 3B, the abundances of the bacterial families *Ilumatobacteraceae*, *Microbacteriaceae*, *Burkholderiaceae*, and *Rhodobacteraceae* were significantly higher after the storm compared to before. The paired *t*‐test analysis revealed *p*‐values of *p* = 0.0216 for *Ilumatobacteraceae*, *p* = 0.0104 for *Microbacteriaceae*, *p* = 0.0229 for *Burkholderiaceae*, and *p* = 0.0499 for *Rhodobacteraceae*, indicating statistically significant increases. In Kirkuk, the three most abundant bacterial genera after the storm were *Flavobacterium* with 1620 reads, *Pseudomonas* with 1249 reads, and *Acinetobacter* with 573 reads. The detailed taxonomic levels of each of these genera are illustrated in Supporting Information S1: Figure S1.

#### 3.1.3 Air Metagenomics Analysis in Baghdad

The metagenomic analysis revealed no countable reads of airborne bacteria from the air filter samples in Baghdad province before the storm. However, as shown in the Pie chart of the Supporting Information S1: Figure S2A after the storm, the most abundant bacterial phyla were as follows: *Proteobacteria* with 6,494 reads (77%), *Bacteroidota* with 764 reads (9%), and *Campylobacterota* with 719 reads (9%) (Supporting Information S1: Figure S2A).

The metagenomic results from the air filter in Baghdad at the genus level after the storm showed that the most abundant bacterial genus was *Fluvibacter*, comprising 45% of the total reads with 3892 reads. Other highly abundant genera included *Comamonas* with 867 reads (10%) and *Aliarcobacter* with 384 reads (4.55%). The distribution of the air microbiome and the taxonomic classification of each bacterium are illustrated in the sunburst chart in Supporting Information S1: Figure S2B.

**Figure 4:**
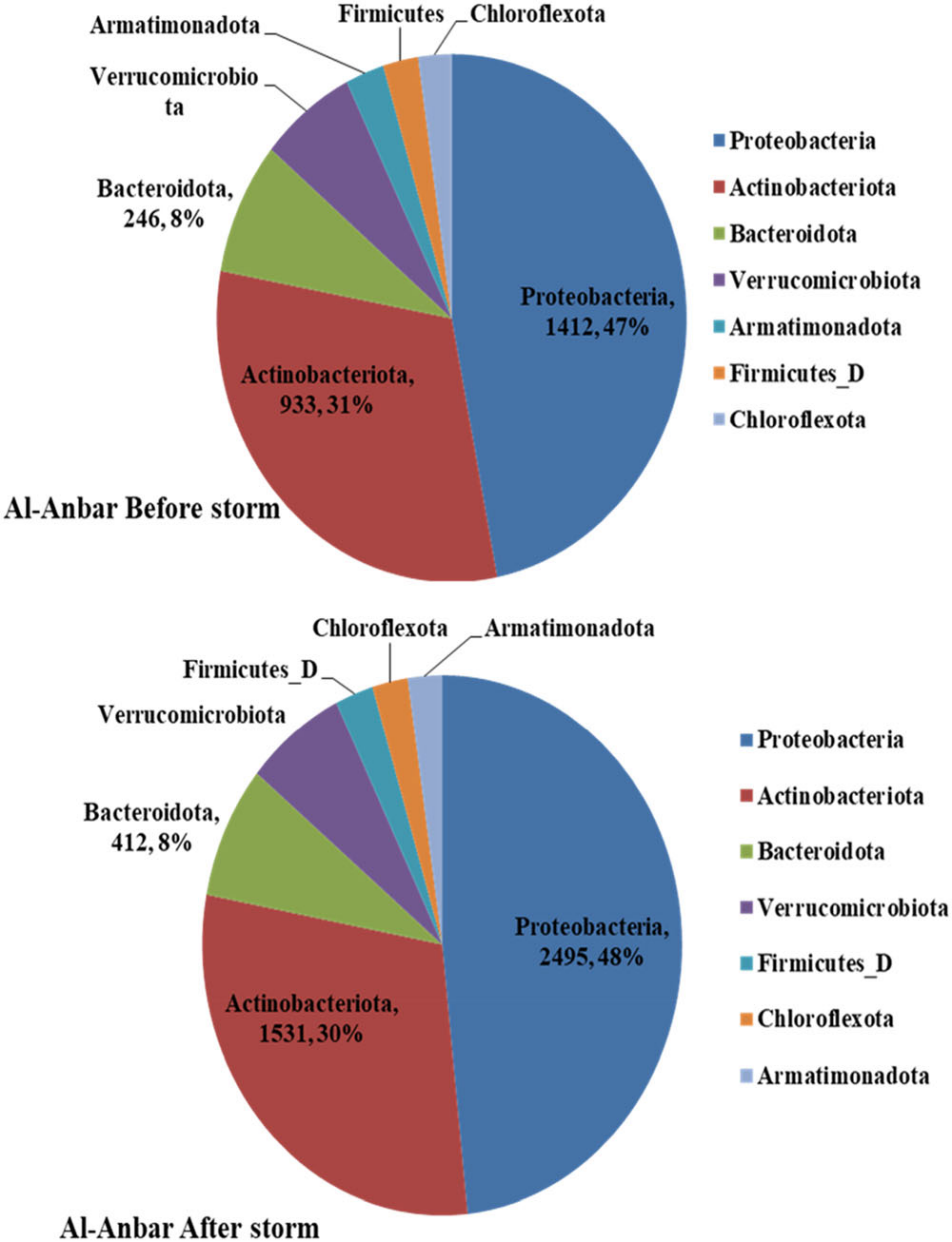
The abundance of bacteria by the phylum level in Al‐Anbar.

At the family level, the most abundant bacterial family was *Rhodocyclaceae* with 4,348 reads (50%). This was followed by *Burkholderiaceae* with 1496 reads (17%) and *Arcobacteraceae* with 387 reads (4.5%), as depicted in the Bar chart of Figure 4.

### 3.2 Comparing Air Metagenomics Analysis in Three Provinces

The abundance percentages of airborne bacteria at the family and genus levels in three provinces are depicted in the bar chart and heat map (Figure 4), respectively. The heatmap graph was utilized to illustrate the variations in airborne bacteria across each sample, employing Euclidean distance metrics to assess microbial diversity among different air filter samples (Shah et al. 2023).

The color intensity in each sample is normalized to indicate its relative abundance among individuals. The heatmap in the Figure 4 uses a color scale from blue to red (values ranging from < 0.2 to 1.618) displayed at the bottom. For example, *Aliarcobacter* shows the highest abundance in the air filter from Baghdad after the storm, represented in red with a Euclidean distance of 1.618.

### 3.3 The Alignment Result and Phylogenetic Tree

The alignment results and corresponding phylogenetic trees for the air microbiome of air filter samples collected from three provinces are depicted in Supporting Information S1: Figure S3. The phylogenetic trees for the three groups were constructed using the “k‐mer mode” (Panyukov et al. 2020).

### 3.4 Alpha Diversity and Estimation of Species Richness in Each Sample

**Figure 5:**
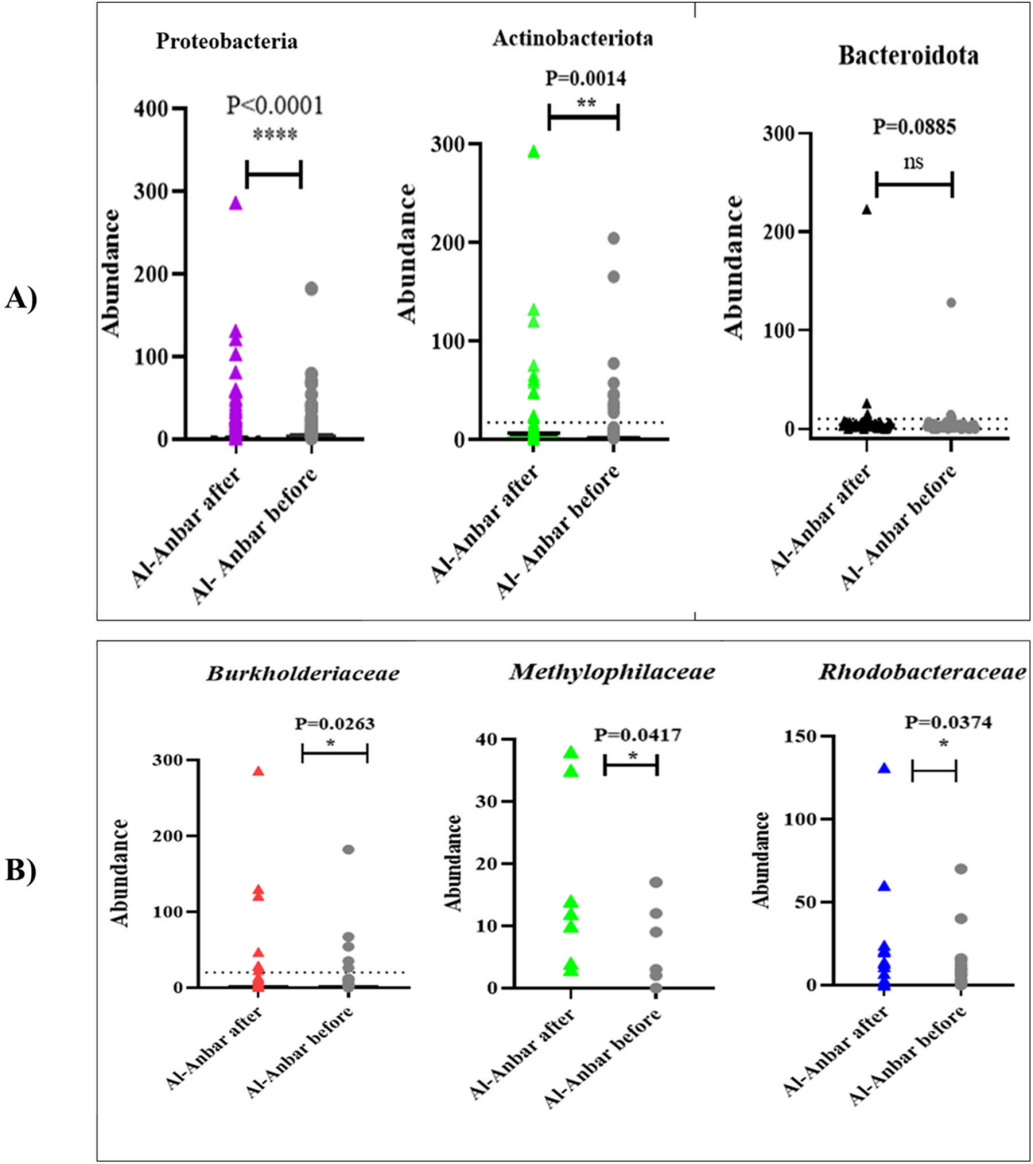
Comparing the bacterial abundance before and after storm in Al‐Anbar: (A) at phylum level; (B) at Family level.

Figure 5 illustrates the Chao1 and Shannon indexes, where the number of reads in each sample is represented by a colored line on the *X*‐axis. The bias‐corrected Chao1 graph indicates that the air filter sample from Al‐Anbar after the storm (represented by the blue cross line) exhibited the highest Chao1 index (310,864), while the sample from Baghdad after the storm (depicted by the red plus line) showed the lowest Chao1 index (98,105), despite having the highest number of reads as shown on the X‐axis (Figure 5). The Shannon entropy analysis also corroborates these findings, with Al‐Anbar after the storm showing the highest Shannon index and Baghdad after the storm showing the lowest. To precise comparison of alpha indexes between different locations, One‐Way ANOVA analysis was employed. The results indicated no significant differences in the diversity of microbial communities from Al‐Anbar before and after the storm. However, the alpha diversity in Al‐Anbar after the storm was significantly higher than that in Baghdad and Kirkuk after the storm (*p* < 0.0001), as illustrated in Figure 5.

### 3.5 Beta Diversity and Principles Component Analysis (PCA)

PCA was utilized to assess the phylogenetic distances between bacterial communities in air filter samples collected from three provinces. The scatterplot generated from PCA illustrates the percentages of variation in the data for these communities. In Supporting Information S1: Figure S4, bacterial communities from Baghdad, Kirkuk, and Al‐Anbar after the storm are represented by red, gray, and green circle symbols, respectively. The analysis reveals a significant phylogenetic distance between the bacterial communities in air filter samples from Kirkuk and Baghdad compared to those from Al‐Anbar after the storm.

### 3.6 Allergic Inflammation Assay After Dust Storm

**Figure 6:**
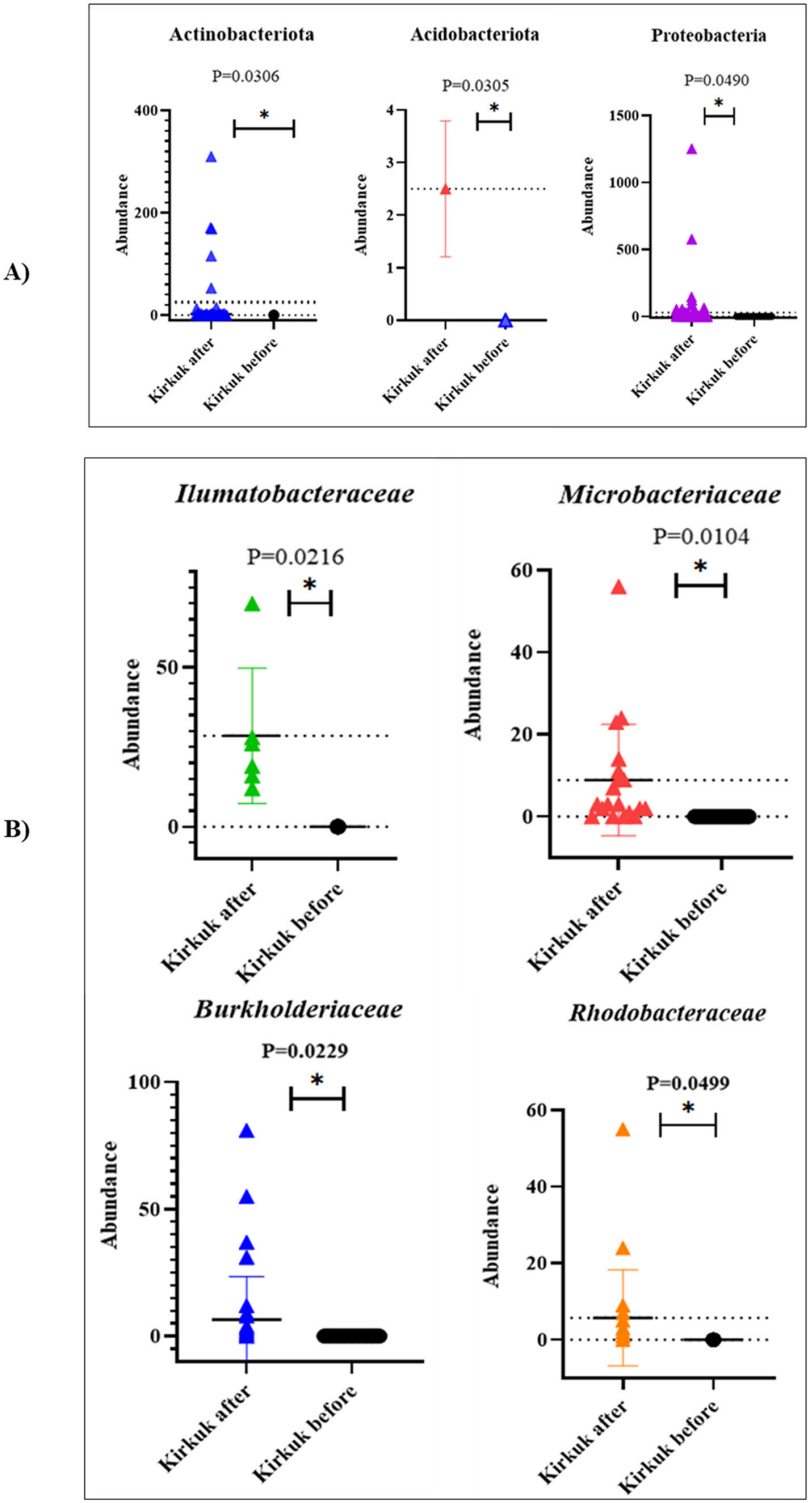
Comparing the bacterial abundance before and after storm in Kirkuk: (A) at phylum level; (B) at Family level.

The inflammation assay of allergy after the dust storm in Al‐Anbar showed that the level of IL‐4 in patients with asthma was significantly greater than before the dust storm (*p* < 0.0001). In the control group, there was no significant difference in the level of IL‐8 (*p* = 0.324), but in the asthmatic group, IL‐8 was not only significantly greater after the dust storm (*p* < 0.0001) but also significantly greater compared to the control group (*p* < 0.0001****) (Figure 6A).

In Kirkuk, IL‐4 and IL‐8 in the control group did not alter significantly following the dust storm (*p* = 0.374 and *p* = 0.229, respectively). In asthma patients, there were, however, significantly greater levels of both cytokines following the storm (*p* < 0.0001), and Tukey's multiple comparison test indicated significantly greater levels in asthma patients compared to controls (*p* < 0.0001) (Figure 6B).

Similarly, in Baghdad, IL‐4 and IL‐8 in asthma patients were considerably greater after the dust storm (*p* < 0.0001), but not in the control group (IL‐4: *p* = 0.575; IL‐8: *p* = 0.117). One‐way ANOVA revealed cytokine levels to be significantly higher in asthma patients compared with the controls (*p* < 0.0001) (Figure 6C).

### 3.7 Comparing the Allergic Inflammatory Response in Three Province

**Figure 7:**
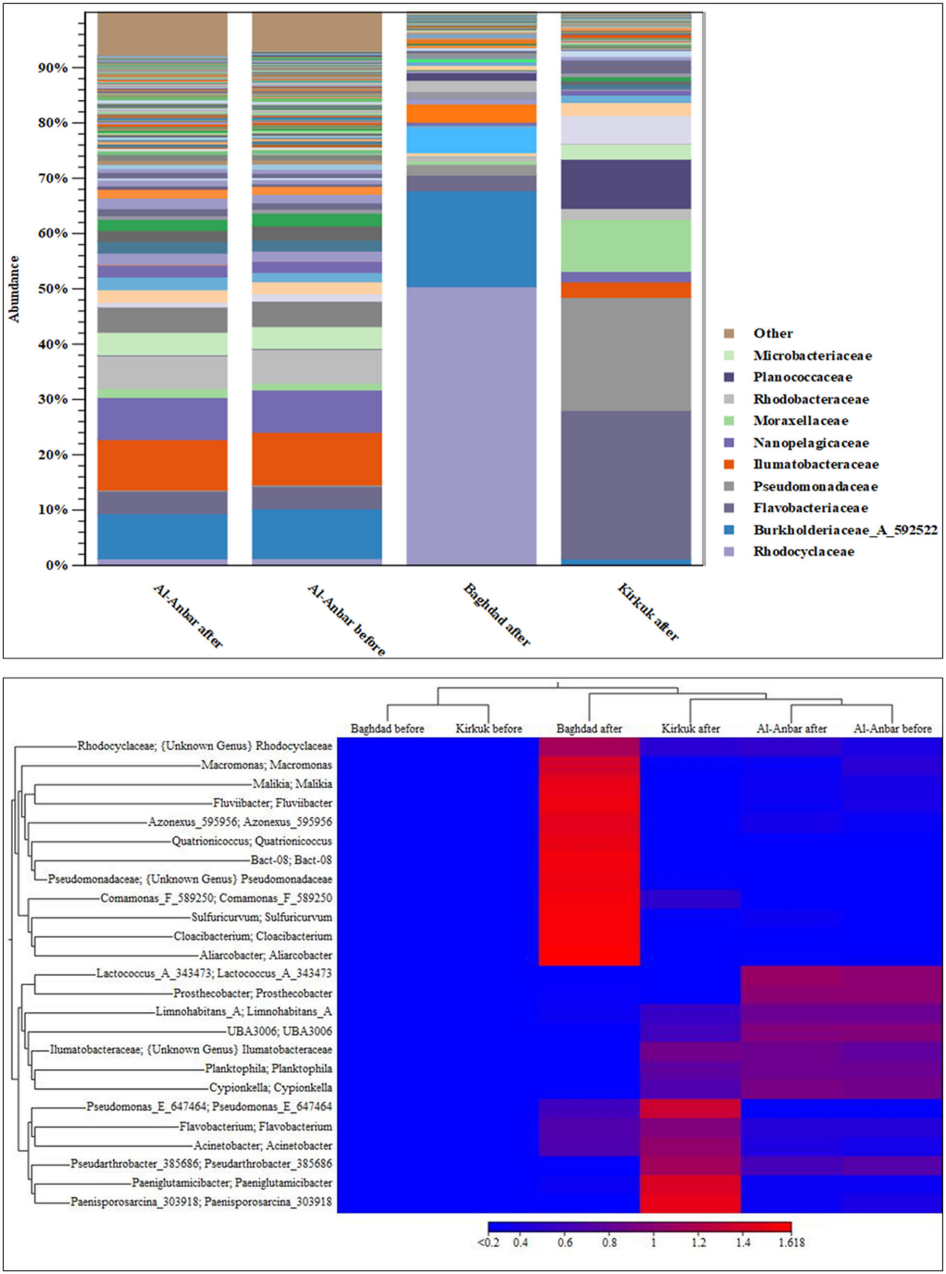
The abundance percentages of airborne bacteria at the family (Bar chart) and genus levels (Heat map) in three provinces.

In the case of the IL‐4 concentration, there was no significant difference between the three areas (Figure 7). According to the One‐way ANOVA and Šídák's multiple comparisons tests, only a significant increase in IL‐8 response was demonstrated in Al‐Anbar province compared to Kirkuk (*p* = 0.006**). The results of multiple comparisons of Al‐Anbar versus Kirkuk, Al‐Anbar versus Baghdad, and Kirkuk versus Baghdad are shown in Figure 7 and Supporting Information S1: Table S1.

### 3.8 Heavy Metals Assay in Air Dust Samples by ICP‐MS

**Figure 8:**
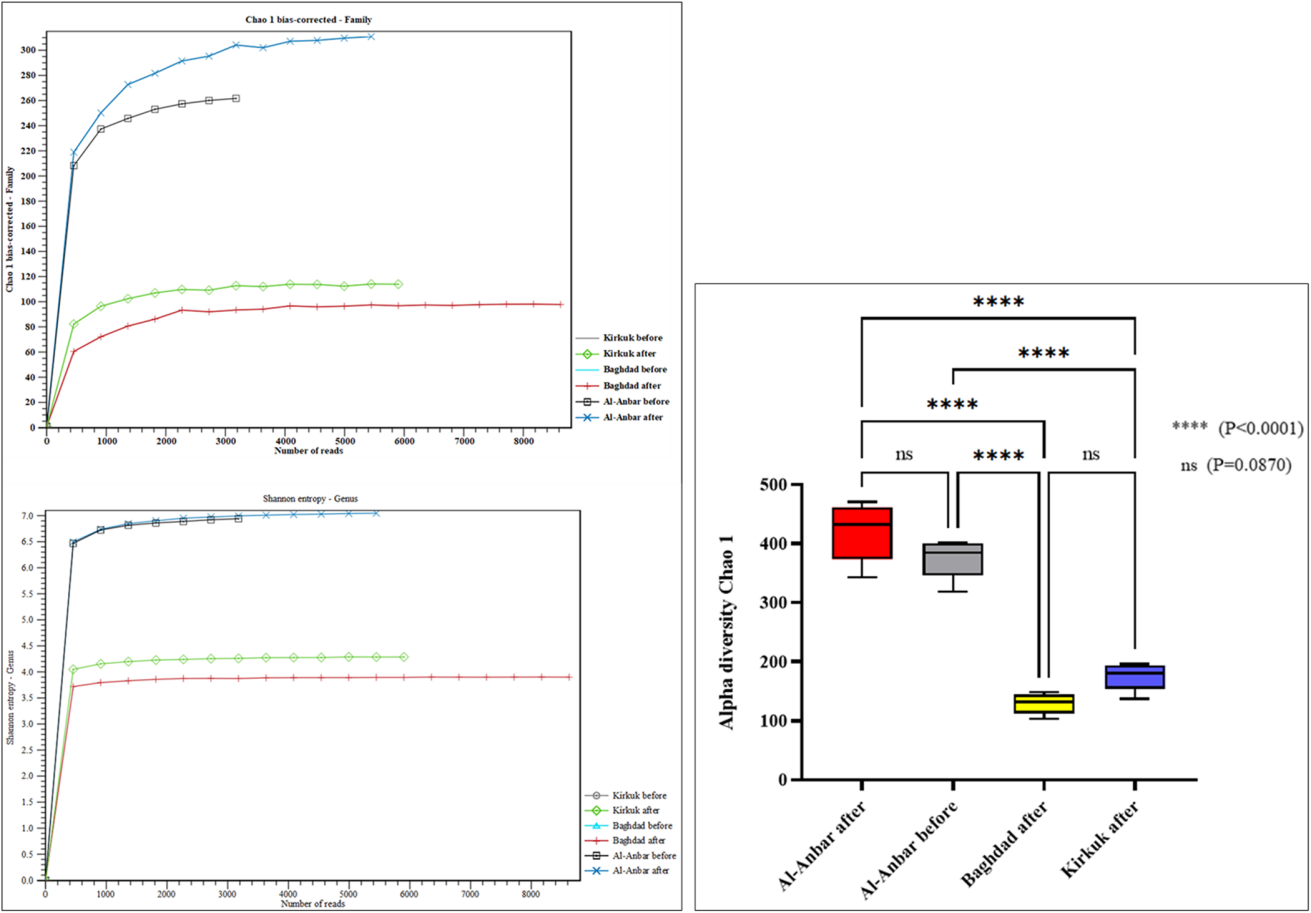
Species richness (Chao1) and diversity (Shannon) estimating.

Due to varying concentrations of metals, we analyzed heavy metals with both low and high concentrations in the air before the dust storm and compared them to levels after the dust storm. The low concentration was from 1 up to 145 µg/kg dry weight, and the high concentration was from 1974 up to 87,321 µg/kg dry weight. Heat map comparisons of the Heavy metals with low and high concentrations in the air samples from three provinces Al‐Anbar, Kirkuk, and Baghdad are shown in Figure 8A,B, respectively. The dark red and dark blue indicates the high concentration, and the light yellow shows the low concentration of elements.

The increased ratio of heavy metals after the dust storm relative to before the storm in the three provinces (Al‐Anbar, Kirkuk, and Baghdad) is shown in Figure 8C. The colored circles, scaled from small to large, represent fold changes: 1/2‐fold (decrease), onefold (no change), and twofold, threefold, and fourfold (increases) in heavy metal concentrations, respectively. In general, Our results showed higher concentrations of As, Ag, B, Ba, Co, Hg, Mg, Mn, Ni, Sn, S, Ti, and V in the air after the dust storm in three provinces of Iraq compared to before dust.

In Al‐Anbar province, there was an increase in concentrations of Sn by fourfolds, B and Ba by 1.5 folds, and S by twofolds after the dusty storm compared to before the storm (Figure 8C). In Kirkuk, There was an increase in concentrations of As by 1.5‐folds; Ag, Co, and S by twofolds; Ni, Ti, and V threefolds, and Ca fourfolds. In Baghdad, There was an increase in concentrations of Ni by sixfolds, As, Co, Mn by 1.5 folds, Mg, and S by 2.5 folds (Figure 8C).

The concentrations of Al, Cd, Cr, Cu, Fe, Hg, Pb, Sn, and Zn did not change after the dust (Figure 8C).

## 4 Discussion

### 4.1 The Cytokine Assay Before and After Storm

Asthma is characterized by chronic airway inflammation, and interleukins like IL‐4 and IL‐8 are involved in the inflammatory and immune responses. Elevated IL‐4 levels are associated with Th2‐driven inflammation, which is common in asthma. IL‐8 is involved in attracting and activating immune cells, including neutrophils, which can contribute to inflammation (Ordoñez et al. 2000). Asthma often has an allergic component. IL‐4 is associated with allergic responses, particularly in promoting IgE production, a key antibody in allergic reactions. Asthmatic individuals often have increased sensitivity to allergens, which can trigger elevated IL‐4 levels (Neis et al. 2006). Genetic predispositions and exposure to environmental factors like air pollution, allergens, respiratory infections, and occupational exposures can influence cytokine levels in individuals with asthma (Mukherjee and Zhang 2011).

This study showed the significantly higher concentration of the IL‐4 and IL‐8 in the Asthmatic patients compared to the control group. These results are in accordance with previously reported studies, where air pollution has been found to be linked with increased cytokine release (Zahedi et al. 2022; Rodríguez‐Cotto et al. 2013) Zahedi et al. 2022 demonstrated a significant increase of IL‐4 in the children of industrial areas, and indicated that they were prone to asthma (Zahedi et al. 2022). Asadi et al in 2014 reported significantly increased serum levels of IL‐4 in the subjects exposed to ambient dusty air pollution compared to the individuals in the clean air condition (Gheybi et al. 2014). They aimed to investigate the link between dusty air pollution and the risk of allergic diseases in the southwestern provinces of Iran. They assessed the cytokine profile and showed that ambient dusty air pollution is considered a high risk for allergic diseases in that population. Study by Rodríguez‐Cotto in 2013 reported that the African dust storms arriving Puerto Rican Coast promote the secretion of IL‐8 and induce cytotoxicity to human bronchial epithelial cells (Rodríguez‐Cotto et al. 2013).

If IL‐4 and IL‐8 levels are found to be elevated in asthma groups compared to healthy controls in Al‐Anbar province or elsewhere, it suggests a dysregulation of the immune response, potentially contributing to the development and exacerbation of asthma in those individuals. However, reasons for elevated IL‐4 and IL‐8 levels in asthma patients in a particular region like Al‐Anbar province would require further investigation.

Dusty winds can carry allergens like pollen, mold spores, and other particles that can sensitize individuals, potentially leading to increased production of IL‐4 and IL‐8 in response to subsequent exposures (Cecchi et al. 2010). Chronic exposure to dusty winds in areas prone to dust storms might result in sustained low‐level inflammation, leading to persistent elevation of IL‐4 and IL‐8 levels in susceptible individuals. This chronic inflammation can have detrimental effects on the airways, especially in individuals with pre‐existing respiratory conditions like asthma (Hajipour et al. 2020).

### 4.2 Pathogenic Dust Bacteria Trigger Asthma Through IL‐4 and Il‐8

The elevated levels of IL‐4 and IL‐8 in asthma patients following dust storms agree with microbial alterations observed in our metagenomic profiling. The samples obtained after the storm are significantly enriched with Proteobacteria (e.g., Pseudomonas, Burkholderiaceae, Rhodobacteraceae) and Actinobacteria in all the study locations, suggesting these microbes cause asthma pathogenesis through established pathways. One of the most important players in this process is lipopolysaccharide (LPS), a Gram‐negative bacterial material found in high concentrations in airborne particulate material (Ren et al. 2019), All epidemiologic studies uniformly link LPS exposure to asthma induction (Pace et al. 2008), especially by virulent organisms such as *Pseudomonas* and *Burkholderia*.

Of note, LPS—not merely dust—is most strongly correlated with decreased lung function (Yang et al. 2020).

This bi‐functional action of this endotoxin's immunomodulation is dose‐dependent as it stimulates Th1 responses at high doses but, with decreased doses (e.g., 100 ng), induces Th2‐dominant allergic reactions with release of IL‐4 (Kim et al. 2007). This Th2 polarization, as observed in our cytokine experiments, exacerbates airway inflammation and asthma (Schuijs et al. 2013; Ma 2020).

Proteobacteria LPS activates Toll‐like receptor 4 (TLR4), triggering inflammatory cascades that enhance IL‐8—a potent neutrophil chemoattractant of asthma exacerbations (Edwards et al. 2012). This is in keeping with our Al‐Anbar findings, wherein Proteobacteria dominance after the storm was associated with higher IL‐8. Similarly, some bacteria are known to polarize dendritic cells to Th2 responses, responsible for the elevated IL‐4 (Medzhitov 2001). Actinobacteria as gram positive bacteria, which were on the rise in Kirkuk, can further contribute to IL‐8 through NLRP3 inflammasome activation caused by peptidoglycan (Keestra‐Gounder and Nagao 2023).

Geographic variation in the composition of microbial communities (e.g., Baghdad's high Fluvibacter levels) would likely have local cytokine profiles. The findings incriminate dustborne bacteria in asthma exacerbation, but future research should isolate individual strains to establish causal mechanisms in controlled settings.

### 4.3 Heavy Metal Assay by ICP‐MS Analysis

In general, our results showed higher concentrations of As, Ag, B, Ba, Co, Hg, Mg, Mn, Ni, Sn, S, Ti, and V in the air after the dust storm in three provinces of Iraq compared to before dust.

Heavy metals such as lead, mercury, cadmium, arsenic, and others can be carried by dusty winds. Inhalation of these heavy metal‐containing dust particles can lead to respiratory issues, exacerbation of pre‐existing respiratory conditions like asthma, chronic bronchitis, and other respiratory diseases (Uduma and Jimoh 2013). Heavy metals are toxic and can accumulate in the body over time, causing both acute and chronic health effects. Chronic exposure to heavy metals from dusty winds may lead to long‐term health issues, including damage to the nervous system, liver, kidneys, and cardiovascular system (Tan et al. 2016).

Children are particularly vulnerable to the adverse effects of heavy metal exposure. Exposure to heavy metals can impact children's growth and development, cognitive function, and lead to learning disabilities and behavioral problems (Al Osman et al. 2019).

Dust containing heavy metals can settle on the ground and contaminate soil and water sources. This contamination can further impact agriculture, food supply, and drinking water sources, posing a risk to both human health and the environment (Mohammed et al. 2011). Individuals who work outdoors or in areas prone to frequent dust storms, such as farmers and construction workers, are at increased risk of exposure to heavy metals in the dusty winds. Occupational exposure to heavy metals can lead to various health issues, including respiratory and skin problems (Han et al. 2020).

Heavy metals can enter the food chain through contaminated soil and water. Plants can uptake these metals, which then accumulate in animals and humans who consume contaminated plants or animals, leading to potential health risks (Ali and Khan 2019).

The combined findings of our Iraqi study and similar regional studies by Soleimani‐Sardo et al. (2023), Zhang et al. (2022), and Moghtaderi et al. (2020) illustrate central patterns in dust‐borne heavy metal contamination and associated health risks in diverse settings. Our investigation of three Iraqi provinces documented the massive post‐storm nickel (sixfold in Baghdad), arsenic, and sulfur elevations, demonstrating the selective mobilization of metals from local soils by dust storms. These acute exposure patterns contrast with Soleimani‐Sardo et al.'s findings in Iran's Jazmurian basin, where chromium and zinc dominated in dust composition, with chronic carcinogenic and ecological risks through multiple routes of exposure (Soleimani‐Sardo et al. 2023).

Other regional comparisons highlight important environmental distinctions. While our Iraqi data showed metal concentration peaks with storms, Zhang et al.'s Nanjing (Zhang et al. 2022), China, study found decreases in chromium, copper, nickel, and lead concentrations during dust events—apparently due to dilution of urban pollution by long‐range transported dust. Notably, both studies implicated nickel as biologically significant, but through different mechanisms: we involved it in asthma exacerbation, while Zhang et al. involved it in direct lung cell toxicity. Their finding of PM2.5 being more cytotoxic but equally inducing inflammatory responses as PM10 confirms our results of metal‐specific immune responses (Zhang et al. 2022). The contrast between outdoor and indoor exposure becomes prominent when our results are contrasted with Moghtaderi et al.'s study of classroom dust in Shiraz, Iran. While we found acute respiratory risks from inhalable, fine metal particulate in storms, Moghtaderi et al. reported persistent lead and iron in classroom dust with no asthma link, which they explained as due to larger particle sizes that would limit lung penetration. The difference points out how exposure context necessarily alters health risk—outdoor storms pose immediate inhalation risks, while indoor dust can establish more chronic exposure conditions (Moghtaderi et al. 2020).

Our study, and those of Kadhum (2020), and Hassan et al. (2024), provide complementary data regarding heavy metal pollution patterns accompanying Iraqi dust storms. We documented significant post‐storm increases in nickel (sixfold in Baghdad), arsenic, and sulfur in three provinces, demonstrating how dust events preferentially mobilize select metals. They support Kadhum et al.'s findings of cadmium and lead as the prominent contaminants in southern Iraq, although their pollution source analysis had pointed to anthropogenic sources of different metals. Hassan et al.'s work in Erbil had expanded on these patterns of pollution by quantifying health risks, in this case, for children who were more sensitive to exposure to arsenic and chromium. Though all three studies noted arsenic as a chronic contaminant, the risk profiles were regionally varied—our acute exposure levels conflicted with Hassan et al.'s estimates of chronic risk, but both underscored vulnerability of children. The cumulative evidence demonstrate that dust storms in Iraq always increase metal concentrations, but the offending contaminants differ by location based on local geology and human exposures. The findings necessitate local monitoring networks and precautions, especially for sensitive populations during dust storms. The combination of patterns of contamination (current study), pollution sources (Kadhum 2020), and health risk data (Hassan et al. 2024) provides a broad perspective on Iraq's dust‐related environmental health issue.

### 4.4 The Difference in Dominant Bacterial Families Between Normal Air and Dusty Air

The difference in dominant bacterial families between normal air and dusty air primarily lies in the composition and abundance of microbial communities present in these environments. In typical or normal air, the bacterial composition is influenced by factors like local vegetation, human activity, and proximity to water bodies. Bacterial families found in normal air often include those associated with the human skin microbiota, vegetation, and soil. The relative abundance of certain bacterial families is relatively stable and is influenced by the local environmental conditions (Mazar et al. 2016).

Dusty air, especially during dust storms or windy conditions, carries a higher concentration of PM including soil particles, pollen, and organic matter. Bacterial families found in dusty air often include those associated with soil and PM, and these can differ from those commonly found in normal air. In summary, the key difference lies in the source and composition of the particles suspended in the air during dusty conditions. Dust storms lift soil particles, vegetation, and other matter into the air, carrying with them a distinct profile of bacterial families, which can differ from the microbial composition typically found in the local, stable environment of normal air. It's important to note that the specifics of bacterial families can vary based on the region, climate, local geography, and the intensity of the dust event (Mazar et al. 2016).

Scientists consider dust storms to be one of the causes of the global increase in allergic and asthma attacks, chronic breathing, lung problems, and cardiovascular and heart diseases. According to a study, the estimated annual amount of desert dust is 0.5 to 5.0 billion tons, which leads to regional or global air migrations.

Another critical factor in the formation of dust storms is severe drought with inappropriate land use. The onset of droughts in the last decade led to the destruction of many agricultural fields and exposed bare land to wind forces (Cook et al. 2008). With the increase in human activities and climate change, desertification intensifies and contributes to the expansion of drylands and the formation of dust storms. Many people are at risk of sore throat and asthma after the dust storm in Iraq.

Dust storms involve the disturbance and suspension of soil particles into the air due to strong winds. As the dust storm lifts these particles, it carries with it a variety of bacteria, contributing to an increase in their presence in the air (Waters et al. 2020; Kellogg et al. 2004). The significantly higher abundance of the *Burkholderiaceae* in Al‐Anbar, and Kirkuk after storm is in line with study by Azad et al. 2023, in which they showed that the *Burkholderiaceae* family in the environment (soil and water) can easily bring community transmission (Azad et al. 2023). This significant increase can be attributed to several factors. Storms stir up soil and dust, dispersing bacteria into the air, and *Burkholderiaceae* thrive in such disturbed conditions due to their metabolic versatility (Kaltenpoth and Flórez 2020). *Burkholderiaceae* are commonly found in arid and agricultural environments (Prudence 2021), which are often the regions where dust storms occur. These environments provide a natural habitat for these bacterial families, and the disturbance of soil during a dust storm releases them into the air (Al Ashhab et al. 2021).

### 4.5 The Association of Asthma, Elevated Heavy Metal Pollutants and Recent Droughts in Iraq

The observed elevated concentrations of various elements (As, Ag, B, Ba, Co, Hg, Mg, Mn, Ni, Sn, S, Ti, and V) in the air following a dust storm in Iraq may be linked to recent droughts. Droughts can lead to increased soil erosion and desertification, resulting in the suspension of fine PM into the air during dust storms. These particulates can carry a range of heavy metals and elements originating from the arid and semi‐arid regions affected by droughts (D'Amato et al. 2015).

The higher levels of IL‐4 and IL‐8 in the serum of patients after the dust storm suggest a potential correlation with drought‐induced environmental changes. Drought conditions can exacerbate air pollution by lifting PM containing pollutants and allergens, which can subsequently trigger inflammatory responses in the respiratory system, reflected in the elevated cytokine levels (Amarloei et al. 2020; Ortega‐Rosas et al. 2021).

Furthermore, the shift in dominant bacterial families in the air after the dust storm, could be associated with alterations in environmental conditions resulting from droughts. Changes in temperature, humidity, and dust concentrations during droughts can influence the composition and prevalence of airborne bacteria, possibly impacting public health (Keswani et al. 2022).

### 4.6 Dust‐Associated Health Risks: A Streamlined Review With Recent Citations (2019–2024)

This Iraqi dust storm research links outdoor dust exposure to elevated heavy metals (As, Hg), elevated pro‐inflammatory cytokines (IL‐4, IL‐8) in asthmatic individuals, and altered bacterial families (e.g., *Burkholderiaceae*). Yet Gangneux et al. (2020) analyzed French indoor dust and found variable microbial composition (Proteobacteria, Firmicutes) with no evident asthma‐related taxa except reduced *Christensenellaceae* in asthmatics' homes—a potential protective mechanism. While both used high‐throughput sequencing, this study integrated environmental (ICP‐MS) and clinical (ELISA) data, as opposed to Gangneux et al. (2020), who focused on taxonomic profiling. The findings suggest differing risks: acute climate‐driven dust storms as opposed to chronic indoor exposures, both targeting microbial dysbiosis in asthma.

By contrast, Onwusereaka et al. (2024) examined preschool indoor/outdoor dust, relating PM10/CO2 and some microbes (Acinetobacter, Aspergillus) to respiratory symptoms in kids. Although this study focused on acute dust storm effects, Onwusereaka et al. considered chronic urban air pollution. Both, however, indicated microbial dysbiosis as a health risk, suggesting dust control (this study) or indoor pollutant reduction (Onwusereaka et al. 2024).

Luo et al. (2024) examined a single Beijing dust event, using culture‐based methods to detect the presence of antibiotic‐resistant bacteria and increased health risks (Hazard Index). Both studies reported increased bacterial richness during dust events, but this study correlated heavy metals and cytokines with asthma, whereas Luo et al. highlighted the danger of antibiotic resistance, particularly among children. Policy implications were varied: regional dust control (this study) versus government intervention in Chinese cities (Luo et al. 2024).

Roy et al. (2023) measured Asian Dust events in Seoul, monitoring PM‐bound metals (Al, Pb, Cd) and pathogenic bacteria (*Rothia mucilaginosa*) using cancer/non‐cancer risk models. Overlaps exist between pollutant elevation and bacterial transitions, but this study was targeted at asthma‐specific immune responses, while Roy et al. observed broader health risks. Both demanded mitigation, but contextually disparate—Iraqi dust control versus urban PM monitoring in Seoul.

Moghtaderi et al. (2020) examined Iranian classroom dust, with elevated metal concentrations (Fe, Mn, Pb) but no asthma association, which differs from this study's storm effects outdoors. Particle size was proposed to restrict lung penetration in classrooms, highlighting alternate exposure routes.

Maloukh et al. (2023) described urban aerial microbes in Abu Dhabi and reported varied bacteria (e.g., Propionibacterium acnes) without health associations, in contrast to the direct asthma correlations of this study. Both report regional microbial differences but with variations in health consequences.

Cha et al. (2017) investigated Korean Asian Dust events, finding transported bacteria (Bacillus, Arthrobacter) but not correlating with health endpoints. This study expanded such work by connecting dust microbiomes and co‐pollutants to asthma and illustrating dust's twin function as microbial carrier and health threat. Together, these articles emphasize the triple‐threat risks of dust—chemical, microbial, geographic—each with varying significance for vulnerable populations, necessitating targeted public health intervention.

## 5 Conclusion and Future Directions

This study provides compelling evidence to attribute dust storms in Iraq's Al‐Anbar, Kirkuk, and Baghdad provinces to significant environmental and health impacts. The results reveal three highly interrelated phenomena: toxic upticks in airborne heavy metals (primarily arsenic, nickel, and mercury), dramatic increases in pro‐inflammatory cytokines IL‐4 and IL‐8 in asthmatic patients, and dramatic shifts in the structure of airborne bacterial communities—particularly upticks in stress‐resistant lineages Burkholderiaceae and Rhodobacteraceae. These results paint a disturbing portrait of the acute health risk to vulnerable groups, especially those with pre‐existing respiratory conditions, that is caused by climate‐increased dust episodes.

In the future, several research avenues of particular significance become apparent from these results. Firstly, it is of imperative need to experimentally validate the pathogenic potential of microorganisms carried by dust using controlled laboratory experiments. Isolation of dominant bacterial species like Pseudomonas and Burkholderiaceae could reveal their special role in triggering inflammatory responses, preferably using airway epithelial cell culture models or murine models of exposure. Second, institute longitudinal surveillance programs to track the seasonal pattern in the dust composition and correlate such changes with hospital admission records of respiratory diseases. Surveillance by such a monitoring program could identify particularly toxic seasons that require public health interventions.

Third, sophisticated geochemical fingerprinting methods should be used to identify locally resuspended pollutants versus long‐range transported dust particles. This identification is important for establishing region‐specific mitigation strategies tailored to the local contamination profile. Fourth, intervention studies are required to assess the effectiveness of control measures like enhanced in‐home and school air filtration systems, or the distribution of particulate‐filtering masks to outdoor workers for dust storm seasons.

Lastly, the larger climatic context must be explored. Future studies should look at how Iraq's growing frequency of droughts and changing land cover trends affect both the severity of dust storms and the poisonousness of their particulate loads. Combining climate modeling with satellite remote sensing could allow the forecasting of dust storm paths and their resultant health effects. Their resolution will not only propel scientific understanding but also inform concrete public health policy to protect exposed populations from dust‐induced respiratory risk.

## Author Contributions

**Alaa Al‐Husseini:** writing – original draft, investigation, formal analysis, resources. **Majid Komijani:** project administration, writing – review and editing, visualization, validation, supervision, methodology, conceptualization. **Rulla Sabah:** investigation, formal analysis, data curation.

## Ethics Statement

The authors have nothing to report.

## Conflicts of Interest

The authors declare no conflicts of interest.

## Supporting information


**Figure S1:** Sunburst diagram of Kirkuk's post‐storm air microbiome: Dominant genera: *Flavobacterium* (1,620 reads), *Pseudomonas* (1,249), and *Acinetobacter* (573), showing their taxonomic hierarchy and relative abundance**. Figure S2:** Post‐storm air microbiome composition in Baghdad: (A) Phylum level: Proteobacteria dominated (77%, 6,494 reads), followed by Bacteroidota (9%, 764 reads) and Campylobacterota (9%, 719 reads). Pre‐storm samples yielded no detectable reads. **(B) Genus level:** *Fluvibacter* (45%, 3,892 reads) was most abundant, with *Comamonas* (10%) and *Aliarcobacter* (4.55%) as secondary taxa. Sunburst chart illustrates taxonomic hierarchy. **Figure S3:** Phylogenetic trees of air microbiome in Al‐Anbar, Kirkuk, and Baghdad: Trees were constructed using k‐mer analysis, revealing taxonomic relationships among bacterial communities across provinces. Branch lengths indicate genetic divergence, with clustering patterns reflecting regional microbiome composition. **Figure S4:** PCA of post‐storm airborne bacterial communities across three provinces: The scatterplot visualizes phylogenetic distances (Bray‐Curtis) between communities: Baghdad (red), Kirkuk (gray), and Al‐Anbar (green). Significant separation was observed between Kirkuk/Baghdad and Al‐Anbar communities, reflecting distinct microbiome compositions. Axes show percentage variation explained.

**Table S1:** Multiple comparisons test of IL‐8 Assay after dust storm in Anbar, Kirkuk, and Baghdad provinces.

## Data Availability

The data that support the findings of this study are available on request from the corresponding author. The data are not publicly available due to privacy or ethical restrictions.
