# Supplementary material for: Impact of Dust Storms on Airborne Bacteria, Heavy Metals, and Inflammatory Markers in Asthmatic Patients
Source: Microbiologyopen. 2025 Nov 4;14(6):e70109. doi: 10.1002/mbo3.70109 (PMC12583928; doi:10.1002/mbo3.70109)
Supplement: Supplementary file 1 — Figure S1: Sunburst diagram of Kirkuk's post‐storm air microbiome: Dominant genera: Flavobacterium (1,620 reads), Pseudomonas (1,249), and Acinetobacter (573), showing their taxonomic hierarchy and relative abundance. Figure S2: Post‐storm air microbiome composition in Baghdad: (A) Phylum level: Proteobacteria dominated (77%, 6,494 reads), followed by Bacteroidota (9%, 764 reads) and Campylobacterota (9%, 719 reads). Pre‐storm samples yielded no detectable reads. (B) Genus level: Fluvibacter (45%, 3,892 reads) was most abundant, with Comamonas (10%) and Aliarcobacter (4.55%) as secondary taxa. Sunburst chart illustrates taxonomic hierarchy. Figure S3: Phylogenetic trees of air microbiome in Al‐Anbar, Kirkuk, and Baghdad: Trees were constructed using k‐mer analysis, revealing taxonomic relationships among bacterial communities across provinces. Branch lengths indicate genetic divergence, with clustering patterns reflecting regional microbiome composition. Figure S4: PCA of post‐storm airborne bacterial communities across three provinces: The scatterplot visualizes phylogenetic distances (Bray‐Curtis) between communities: Baghdad (red), Kirkuk (gray), and Al‐Anbar (green). Significant separation was observed between Kirkuk/Baghdad and Al‐Anbar communities, reflecting distinct microbiome compositions. Axes show percentage variation explained. [file MBO3-14-e70109-s001.docx]

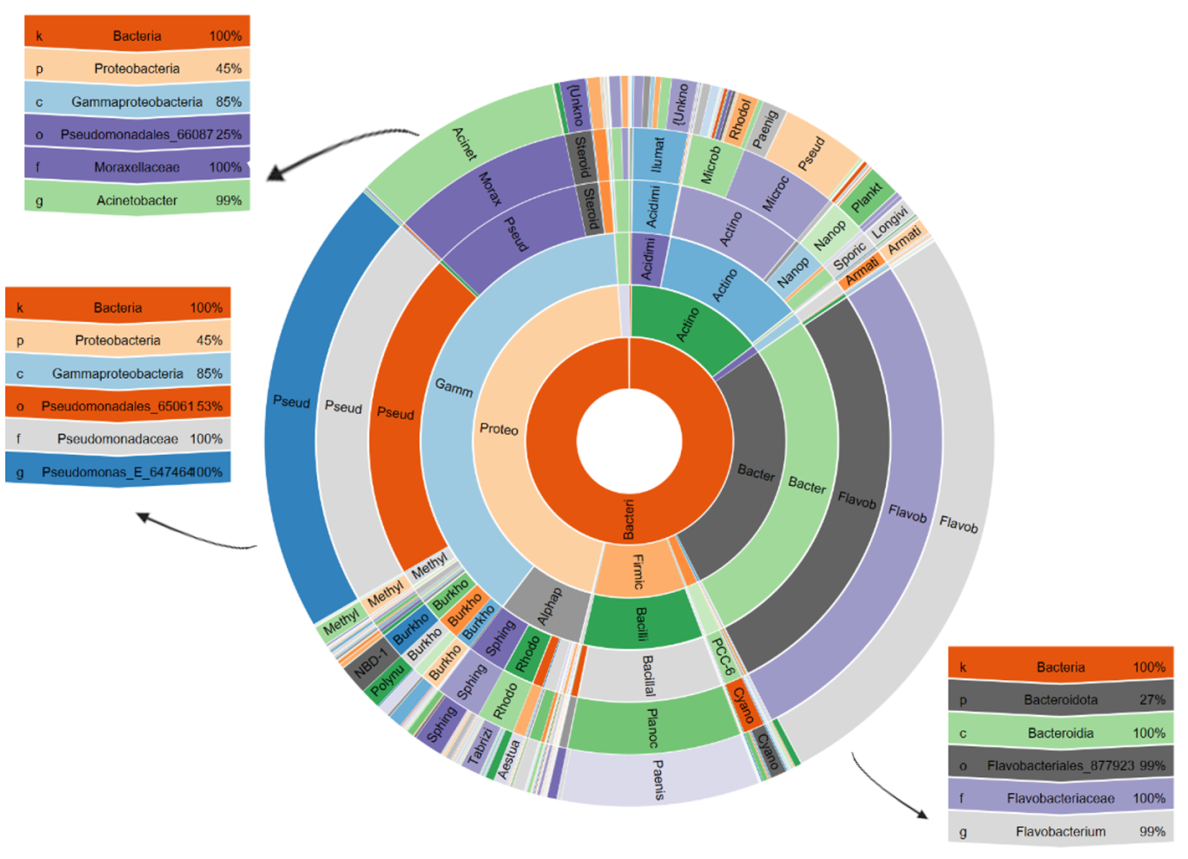


**Figure S1: Sunburst representation of the overall distribution of the Air microbiome in Kirkuk after storm**


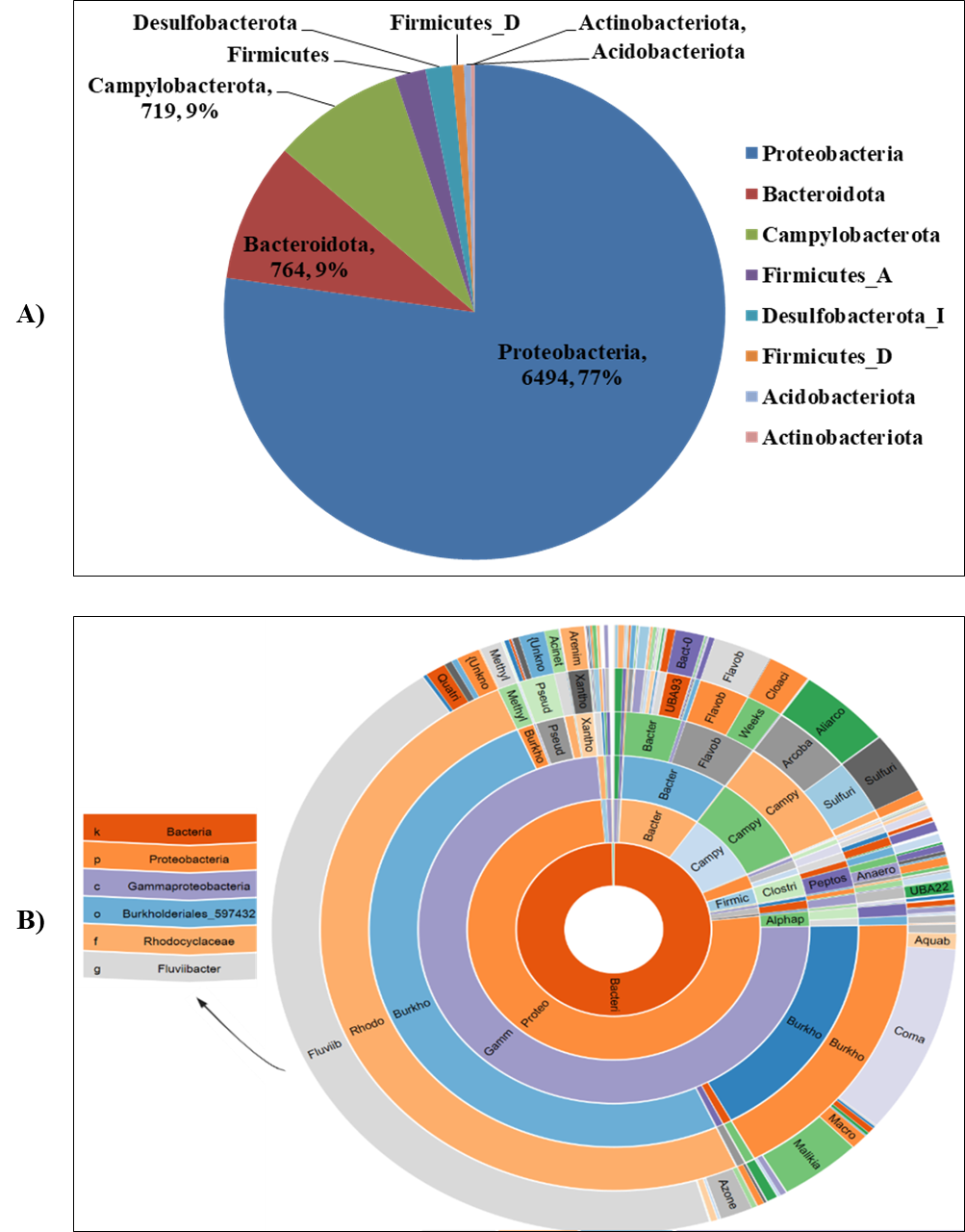


**Figure S2: Air microbiome in Baghdad after storm: A) Pie chart of air bacterial abundance at phylum level B) Sunburst representation of the of air bacterial abundance at Genus level**


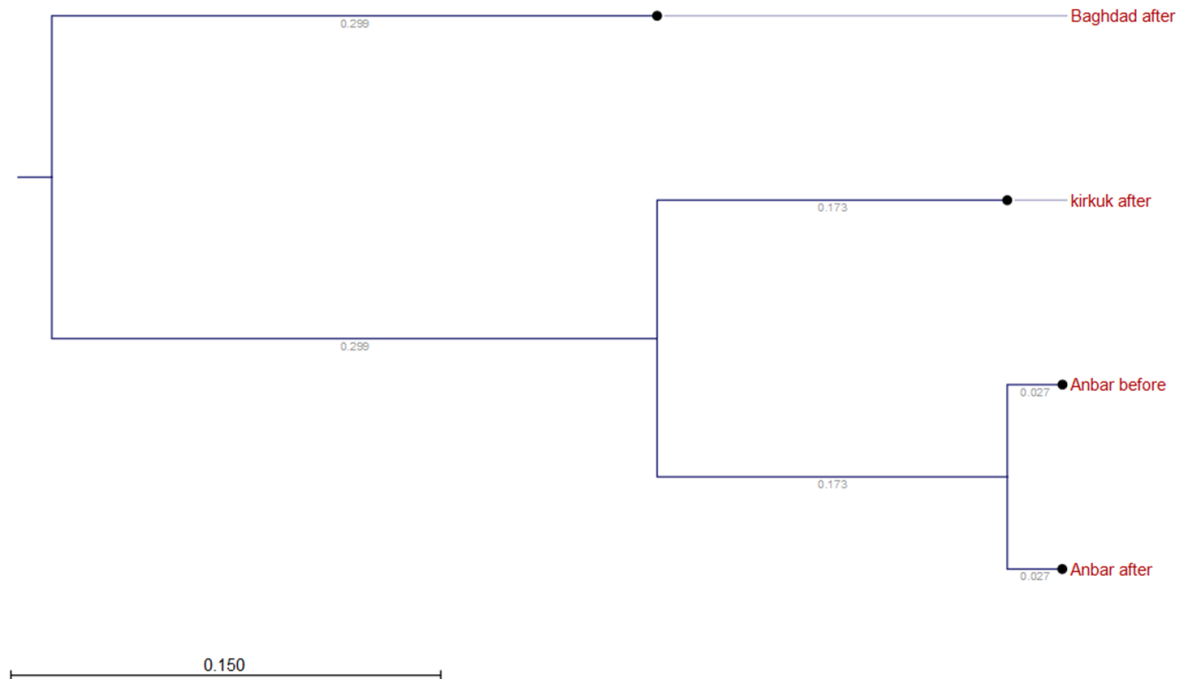


**Figure S3 : The phylogenetic tree corresponding to the air microbiome in Al-Anbar, Kirkuk, and Baghdad**

**
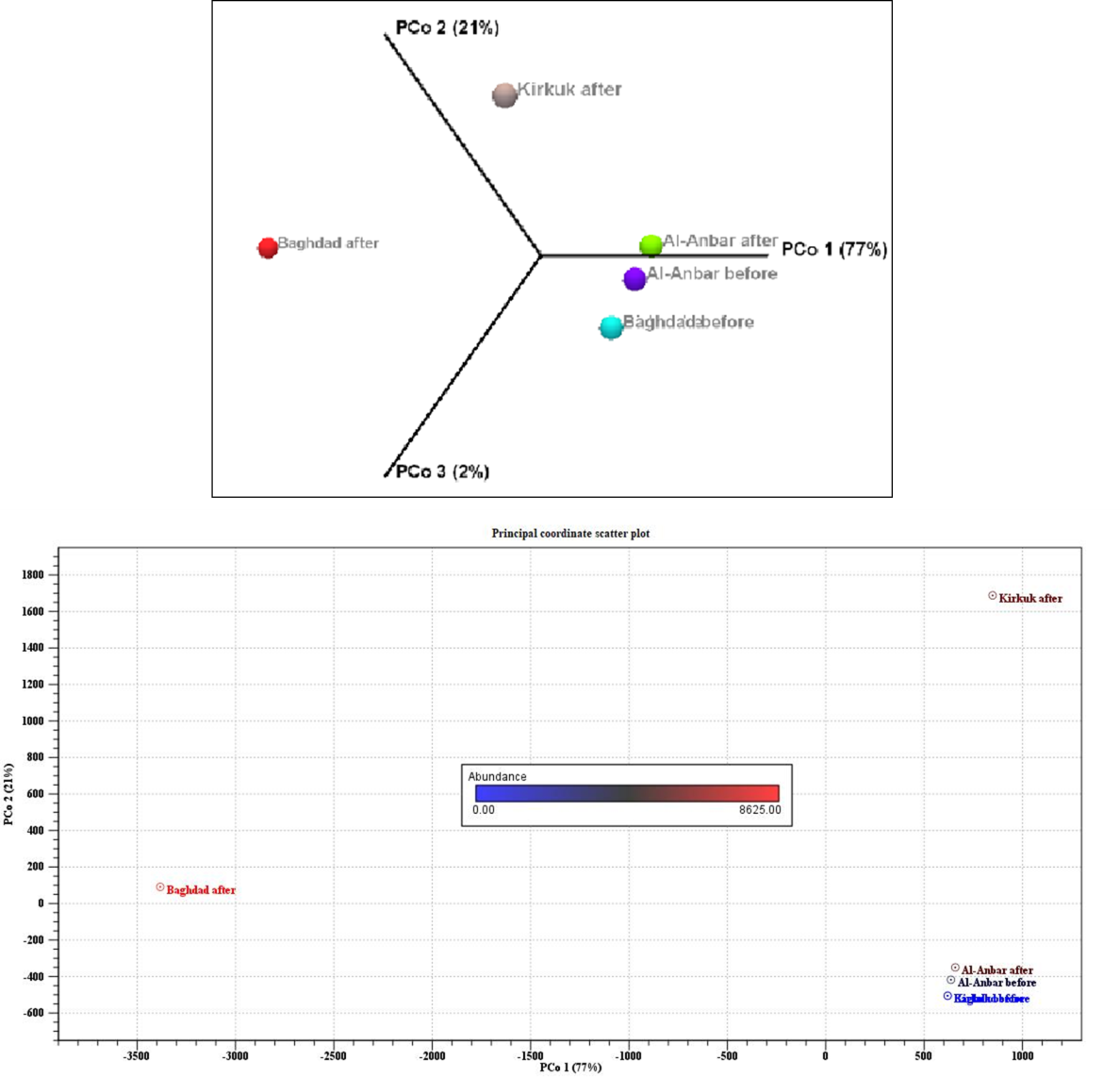
**

**Figure S4: Beta diversity analysis and Phylogenetic distance between groups based on the Bray-Curitis**
