# Supplementary material for: Impact of Dust Storms on Airborne Bacteria, Heavy Metals, and Inflammatory Markers in Asthmatic Patients
Source: Microbiologyopen. 2025 Nov 4;14(6):e70109. doi: 10.1002/mbo3.70109 (PMC12583928; doi:10.1002/mbo3.70109)
Supplement: Supplementary file 2 — Table S1: Multiple comparisons test of IL‐8 Assay after dust storm in Anbar, Kirkuk, and Baghdad provinces. [file MBO3-14-e70109-s002.docx]

**Table S1: Multiple comparisons test of IL-8 Assay after dust storm in Anbar, Kirkuk, and Baghdad provinces**

| **Šídák's multiple comparisons test** | **Mean Diff.** | **95.00% CI of diff.** | **Below threshold?** | **Summary** | **Adjusted P Value** |
| --- | --- | --- | --- | --- | --- |
| **Anbar vs. Kirkuk** | 43.95 | 9.954 to 77.96 | Yes | ** | 0.0068 |
| **Anbar vs. Baghdad** | 28.07 | -6.224 to 62.36 | No | ns | 0.1410 |
| **Kirkuk vs. Baghdad** | -15.89 | -50.18 to 18.41 | No | ns | 0.5987 |
